# Fabrication of SiC Porous Ceramics by Foaming Method

**DOI:** 10.3390/ma16041342

**Published:** 2023-02-04

**Authors:** Jing Zhao, Xiaoqi Ban, Yifan Yang, Zhigang Yuan, Hongqiang Ru, Desheng Su

**Affiliations:** 1 School of Materials Science and Engineering, Shenyang Ligong University, Shenyang 110159, China; 2 Liaoning Ultra High Power Graphite Electrode Material Professional Technology Innovation Center, Dandong 118100, China; 3 Liaoning Xiyuan Graphite Technology Co., Ltd., Tieling 112703, China; 4 Key Laboratory for Anisotropy and Texture of Materials of Ministry of Education (ATM), Northeastern University, Shenyang 110819, China

**Keywords:** SiC, porous ceramics, foaming, hierarchical, porosity, mechanical properties

## Abstract

In this work, hierarchically porous SiC ceramics were prepared via the foaming method. Porous ceramics with tunable, uniform, and bimodal pore structures were successfully fabricated in a facile way. The formation mechanisms of the 1st and 2nd modal macropores are the H_2_O_2_ foaming process and SiC particle overlap, respectively. The effect of pore-foaming agent amount, foaming temperature, and surfactant was investigated. According to the results, with increasing H_2_O_2_ amount, the porosity, pore size, and interconnectivity of the 1st modal pores increased, whereas bulk density and strength decreased. The porosity increased while the strength decreased as the foaming temperature increased. Surfactants increased pore interconnectivity and porosity. When the foaming temperature was 85 °C, and the addition of H_2_O_2_ was 5 wt.%, the porosity, bulk density, flexural strength, and compressive strength were 56.32%, 2.8301 g/cm^3^, 11.94 MPa, and 24.32 MPa, respectively. Moreover, SiC porous ceramics exhibited excellent corrosion resistance to acids and alkalis.

## 1. Introduction

SiC porous ceramics are a kind of “green functional material”. Due to their high porosity, low bulk density, large specific surface area, good permeability, easy cleaning and regeneration, high temperature resistance, thermal shock, corrosion, and mass loading [1,2,3,4,5], SiC porous ceramics have shown promising applications in many fields, such as catalyst carriers, gas-liquid filtration, thermal insulation, biomaterials, and sensors [6,7,8,9,10].

The primary characteristic of porous ceramics is their porous structure, which has a significant impact on their applications. In recent years, there has been an increasing demand for the versatility of porous materials, resulting in the development of multi-scale and multi-level porous structures. Due to their ability to perform multiple tasks simultaneously, such materials are widely used in catalytic carriers, high-temperature gas filtration, supercapacitors, porous burners, etc. [11,12,13,14]. Therefore, to achieve versatility, it has been necessary to develop porous structures with both high porosity and different pore sizes.

Currently, the fabrication of porous ceramics with hierarchical porous structures typically requires multiple steps, such as introducing micro/mesostructures into a preformed macroporous skeleton structure. Accordingly, in addition to the preparation of the macroporous skeleton, several other processes are employed to introduce the hierarchically porous structure, including the growth of one-dimensional nanostructures [15], coating [16], etching [17], and the use of preceramic polymers [18], which undoubtedly increase the cost and complexity of the preparation process. Therefore, it is still highly desirable to develop porous ceramics with hierarchically porous structures in a simple manner.

In contrast, conventional methods for fabricating SiC porous ceramics, such as replica [19,20], sacrificial template method [21,22], and the foaming method [23], are simpler and easier to perform. Among them, the foaming method, which was invented by Sundermann in 1973 [24], has attracted widespread attention due to its simple and straightforward operation. The basic principle is that inorganic or organic chemical substances (foaming agents) are added to the ceramic components to generate volatile gas bubbles by physical, chemical, and mechanical stirring methods, which are then dried and sintered to produce porous ceramics. However, it is primarily suitable for preparing porous ceramics with closed pores. During the foaming process, drainage of the liquid film can lead to foam instability and aggregation [25], resulting in non-uniform pore size and blank collapse. To solve this problem, a three-dimensional network gel is formed by introducing organic monomers into an in-situ polymerization reaction, allowing the ceramic foam slurry to be rapidly cured into a porous ceramic blank with high strength [26,27,28]. Han [29] fabricated Si_3_N_4_/SiC porous ceramics by foam-gelcasting with a porosity of 68.54 ± 0.73%. The flexural and compressive strengths were 5.28 ± 0.17 MPa and 12.86 ± 1.55 MPa, respectively. Wu [30] prepared porous ceramics with porosity up to 80.1% by the gelcasting method. However, the porous ceramics fabricated by the foaming method only have monomodal porous structures, which does not allow for the preparation of hierarchically porous structures. It is expected that SiC porous ceramics with hierarchically three-dimensional interconnected open porous structures can be prepared by the foaming method.

In this work, hierarchically porous SiC ceramics with combined structures and functions were prepared by the foaming method. These ceramics have a uniform porous structure, controllable pore size, low cost, and high strength. The effect of pore-foaming agent amount, foaming temperature, and SDS was investigated in detail.

## 2. Materials and Methods

### 2.1. Materials

Commercial α-SiC powders (*d*_50_ = 0.45 μm, 99% purity) were purchased from Yonghao silicon carbide micro powder Co. Ltd., Weifang, China. Acrylamide (AR), N’N’-methylenebisacrylamide (AR), ammonia solution (25 wt.%), H_2_O_2_ (30 wt.%), and sodium dodecyl sulfate (AR) were purchased from Sinopharm Chemical Reagent Co. Ltd., Shanghai, China. Sucrose (CR) was purchased from Sigma-Aldrich (St. Louis, MO, USA) [31]. All chemicals were used as received.

### 2.2. Preparation of SiC Porous Ceramics

Firstly, the raw materials SiC, acrylamide, N’N’-methylenebisacrylamide, sucrose, ammonia solution, and deionized water were mixed by ball milling at a mass ratio of 250:12.5:1:25:3.75:72.5 for 4 h. Nylon ball milling jars with grinding media of Al_2_O_3_ balls were used, and the speed was 20 r/min. Then, a certain mass (5 wt.%, 7.5 wt.%, 10 wt.%, 12.5 wt.%, 15 wt.% relative to the mixed SiC slurry) of hydrogen peroxide (H_2_O_2_) and sodium dodecyl sulfate (SDS, surfactant) was added. After mixing uniformly, the mixed slurry was poured into a mold, foamed, and dried at 75 °C, 85 °C, and 95 °C, respectively. Finally, SiC porous ceramics were sintered at 2000 °C under an Ar atmosphere with a heating rate of 5 °C/min and a holding time of 30 min.

### 2.3. Properties Testing and Characterization

The morphology and microstructure of SiC porous ceramics were analyzed by scanning electron microscope (SEM) (JEOL JSM-6510A, Tokyo, Japan). The physical phase analysis was performed by X-ray diffractometer (X’Pert Pro NPP, Panalytical, Almelo, The Netherlands), with a Cu target (accelerating voltage and current intensity of 45 kV and 40 mA, respectively), and scanning angle (2θ) of 10~90° with a scanning step of 0.033°.

The bulk density of ceramics was measured using Archimedes’ principle, and water was used as a liquid medium [32,33]. The bulk density was calculated via the following equation:

(1) $$\rho =\frac{m_{1}}{m_{3}-m_{2}}\rho_{H_{2}O}$$

where *m*_1_ is the dry weight of materials (g), *m*_2_ is the weight of materials immersed in water (g), *m*_3_ is the weight of materials suspended in water (g), and $\rho_{H_{2}O}$ is the density of water (g/cm^3^).

The porosity was measured by the mass-volume method. The porous ceramic samples were fabricated into regular shapes to measure their size and volume. The porosity was calculated by the following equation:

(2) $$P=\left(1-\frac{m}{V\rho_{S}}\right)\times 100\%$$

where *m* is the mass of samples (g), *V* is the volume of samples (cm^3^), and *ρ*_S_ is the density of SiC dense ceramics (g/cm^3^).

The mechanical properties of materials were measured by the microcomputer-controlled electronic universal testing machine (CMT5105, New Sansi Enterprise Development Co., Shanghai, China) with a loading rate of 1 mm/min. The samples were fabricated in a rectangular shape with a dimension of 5 mm × 10 mm × 10 mm for the strength test.

The corrosion resistance of SiC porous ceramics was evaluated by measuring the loss rate of strength and weight after corrosion by acid and alkali solutions. Herein, the polished samples were put into a corrosive solution with 20 wt.% H_2_SO_4_ or 1 wt.% NaOH, heated to boiling, and held for 1 h. Subsequently, the samples were washed and dried, after which their residual flexural strength and weight loss were measured.

## 3. Results and Discussion

### 3.1. Physical Phase Analysis of SiC Porous Ceramics

**Figure 1:**
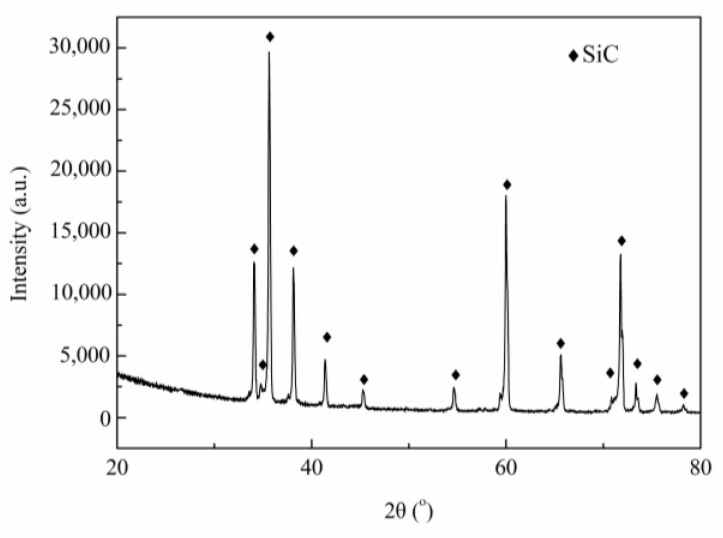
XRD pattern of SiC porous ceramics.

XRD physical phase analysis of sintered SiC porous ceramics was performed, and the results are shown in Figure 1. It can be seen that the primary phase of sintered porous ceramics was SiC, indicating that the organics in the green body had completely decomposed after high-temperature sintering.

### 3.2. Effect of H_2_O_2_ Addition on the Pore Structure of SiC Porous Ceramics

**Figure 2:**
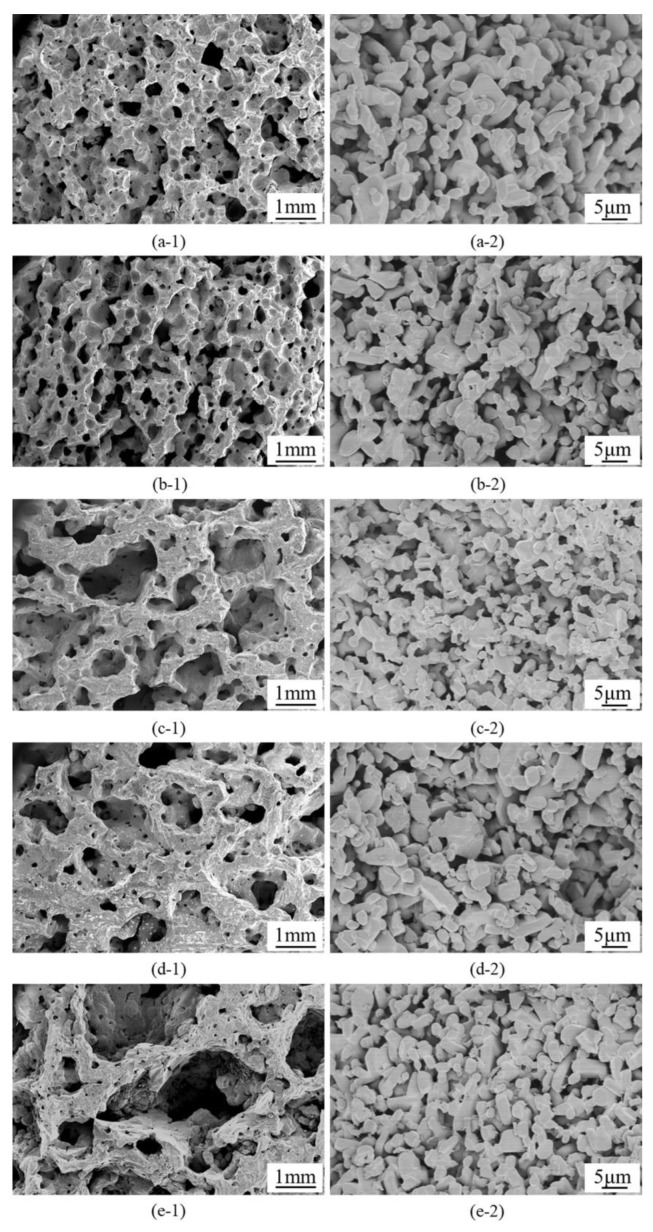
SEM images of SiC porous ceramics prepared with different H_2_O_2_ additions: (**a-1**) 1st pore morphology with 5 wt.% H_2_O_2_; (**a-2**) 2nd pore morphology with 5 wt.% H_2_O_2_; (**b-1**) 1st pore morphology with 7.5 wt.% H_2_O_2_; (**b-2**) 2nd pore morphology with 7.5 wt.% H_2_O_2_; (**c-1**) 1st pore morphology with 10 wt.% H_2_O_2_; (**c-2**) 2nd pore morphology with 10 wt.% H_2_O_2_; (**d-1**) 1st pore morphology with 12.5 wt.% H_2_O_2_; (**d-2**) 2nd pore morphology with 12.5 wt.% H_2_O_2_; (**e-1**) 1st pore morphology with 15 wt.% H_2_O_2_; (**e-2**) 2nd pore morphology with 15 wt.% H_2_O_2_.

The microstructures of SiC porous ceramics with different H_2_O_2_ additions are shown in Figure 2. Interestingly, there were two series of macropores discernible in the SiC ceramics fabricated by the foaming method, forming unique hierarchically macroporous structures: these are the 1st modal macropores with pore sizes ranging from 0.5 to 3 mm and the 2nd modal pores with pore size approximately 2 μm. To our knowledge, the conventional foaming approach often produces a monomodal porous structure [34,35]. In contrast, SiC porous ceramics with a hierarchically macroporous structure, as reported here, were obtained in a facile way.

**Figure 3:**
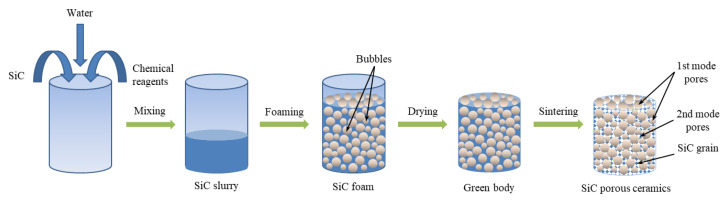
Schematic illustration of the porous structures formation mechanism.

The formation mechanism of these hierarchically porous structures was that H_2_O_2_ decomposed and formed air bubbles, which were fixed in the body by thermally initiated gelation of acrylamide, forming 1st modal pore structures. The 2nd modal pore structures were formed by SiC particles overlapping each other. The porous structure formation mechanism is schematically shown in Figure 3.

The 1st modal pore size was associated with H_2_O_2_ additions. At low H_2_O_2_ content, the 1st modal pores were less uniform and smaller in pore size (Figure 2(a-1)), whereas higher amounts of H_2_O_2_ resulted in porous ceramics with much more uniform porous structures and larger pore sizes (Figure 2(b-1),(c-1)). With the increase of H_2_O_2_ additions, the amount of gas produced in the foaming process increased, leading to an increase in porosity and 1st pore size, as well as an enhancement of pore connectivity. The uniformity of the porous structure decreased as the H_2_O_2_ content further increased to more than 12.5 wt.%, and the pore morphology became irregular (Figure 2(d-1)–(e-1)). H_2_O_2_ additions had no effect on the 2nd modal pore size (Figure 2(a-2)–(e-2)).

**Figure 4:**
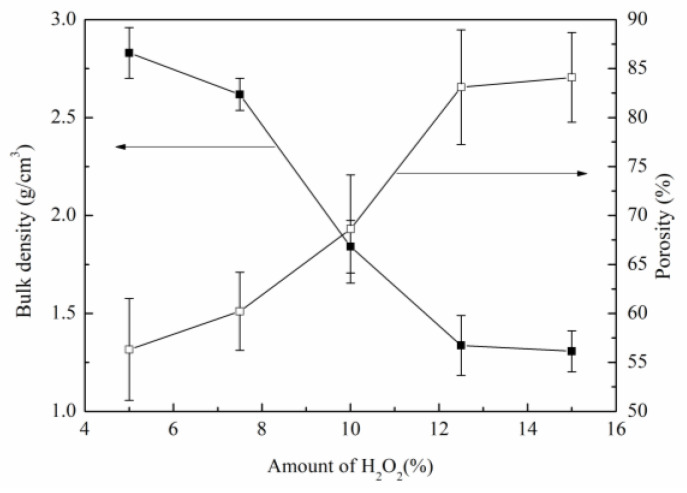
Effects of H_2_O_2_ additions on porosity and bulk density of SiC porous ceramics.

Figure 4 shows the effect of H_2_O_2_ additions on porosity and bulk density. It can be seen that, with the increase of H_2_O_2_ additions from 5 wt.% to 12.5 wt.%, the foaming process became more intense, and the volume of gas produced increased, which led to a decrease in bulk density and an increase in porosity in the SiC porous ceramics. However, when more than 12.5 wt.% H_2_O_2_ was added, the number and volume of bubbles increased dramatically due to the excessive gas generated, which led to the phenomenon of gas overflow and bubble merging, resulting in a non-obvious increase in porosity. The maximum porosity of 84.1% was obtained when H_2_O_2_ was added at 15%, where the bulk density was 1.3068 g/cm^3^.

**Figure 5:**
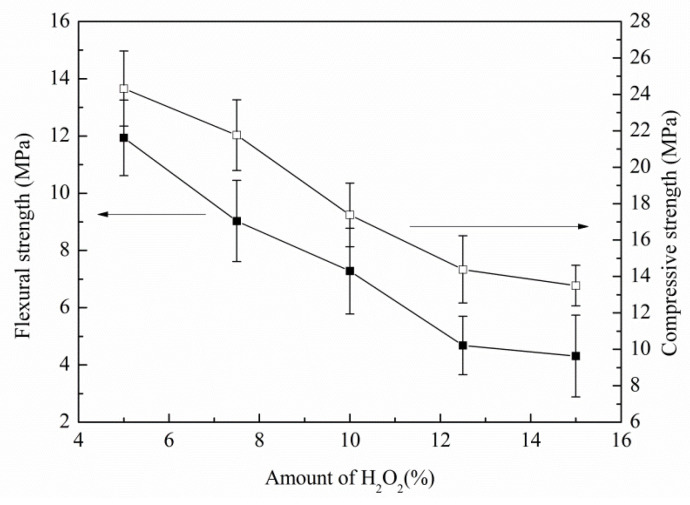
Effects of H_2_O_2_ additions on flexural strength and compressive strength of SiC porous ceramics.

Figure 5 shows the effect of H_2_O_2_ additions on the mechanical properties of SiC porous ceramics. It was demonstrated that, with increasing H_2_O_2_ content, due to its increased porosity, the internal structure gradually loosened and the relative density decreased, leading to a decrease in the flexural and compressive strength of SiC porous ceramics. According to Ryskewitsch’s empirical formula [36] and Breny and Green’s research [37], strength is determined by porosity, with high porosity resulting in low strength. When 5 wt.% H_2_O_2_ was added, the mechanical properties of the obtained SiC porous ceramics were optimal, where the porosity, bulk density, flexural strength, and compressive strength were 56.32%, 2.8301 g/cm^3^, 11.94 MPa, and 24.32 MPa, respectively.

Table 1 shows the relationship between H_2_O_2_ amounts and strength loss rate after acid/alkali corrosion. It can be seen that the SiC porous ceramics exhibited excellent resistance to acid and alkali corrosion with a strength loss rate of only 0.83–3.85%. Moreover, as the amount of foaming agent increased, the flexural strength loss rate of SiC porous ceramics after acid and alkali corrosion gradually increased. This was due to their porosity being proportional to the H_2_O_2_ amount, and the high porosity increased the contact area between acid and alkali corrosive agents and SiC porous ceramics, thereby increasing corrosion.

Furthermore, the strength loss rate of the SiC porous ceramics after alkali corrosion was higher than that after acid corrosion, indicating that their resistance to acid corrosion was better than that to alkali corrosion. SiC has difficulty reacting with acids and alkalis, but it finds it extremely easy to react with oxygen to form SiO_2_; therefore, its surface usually has a protective film of SiO_2_. The presence of a SiO_2_ protective film prevents O_2_ from further reaction with internal SiC. Only when this SiO_2_ film is destroyed will the internal SiC continue to be oxidized. In general, sintered samples contain very small amounts of SiO_2_, so they react easily with alkaline substances. In alkaline conditions, SiO_2_ reacts with NaOH as follows:

SiO_2_ + NaOH→NaSiO_3_ + H_2_O (3)

The SiO_2_ film is destroyed, allowing the oxidation of SiC to proceed further, while the resulting SiO_2_ will continue to interact with alkali, and this process will continue in a cyclic manner, resulting in a decrease in the strength of the samples. In contrast, SiO_2_ is an acidic substance that is relatively stable in an acidic environment. Therefore, SiC porous ceramics exhibit different strengths and weight loss rates after acid and alkali corrosion.

### 3.3. Effect of Foaming Temperature on SiC Porous Ceramics

**Figure 6:**
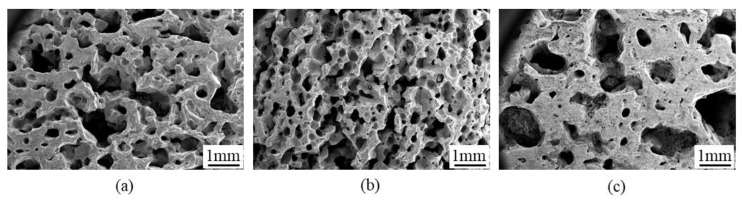
SEM images of SiC porous ceramics prepared at different foaming temperatures with 7.5 wt.% H_2_O_2_ addition: (**a**) 75 °C; (**b**) 85 °C; (**c**) 95 °C.

The microstructures of SiC porous ceramics with different foaming temperatures are shown in Figure 6. As can be seen, both pore uniformity and pore size were affected by the foaming temperature. When foaming at a lower temperature of 75 °C, the gas generation speed was low due to the slow decomposition of H_2_O_2_. Meanwhile, the gelation time was extended, making the bubbles prone to merging and non-uniformity. As the foaming temperature increased to 85 °C, the speed of gas generation and gelation was fast, which allowed a large number of bubbles to be generated and quickly fixed in the blanks before they merged, resulting in the porous structures with uniform and high porosity. When the foaming temperature was further increased to 95 °C, due to the high temperature making H_2_O_2_ violently decompose, a large amount of gas was generated in a short time, resulting in the merging of bubbles and the collapse of blanks, which finally causes non-uniform porous structures.

The properties of the SiC porous ceramics are shown in Table 2. With the increase in foaming temperature, the porosity increased, and the bulk density, flexural strength, and compressive strength decreased. This was due to the higher foaming temperature; the faster foaming agent decomposed in the slurry, resulting in increased porosity and decreased strength.

### 3.4. Effect of Surfactant on SiC Porous Ceramics

**Figure 7:**
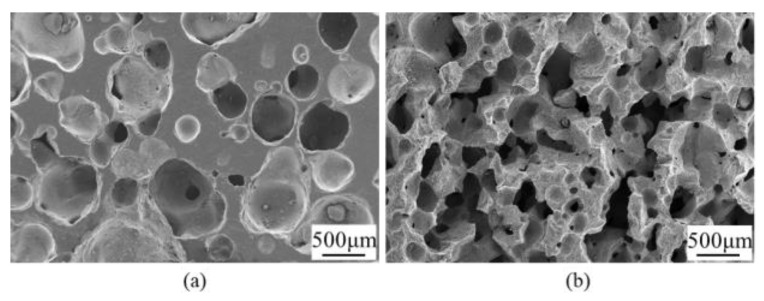
SEM images of SiC porous ceramics with different amounts of SDS: (**a**) 0 and (**b**) 0.1 wt.%.

SEM images of the SiC porous ceramics with different amounts of surfactant (SDS) are shown in Figure 7. As can be seen, the addition of SDS increased the three-dimensional interconnectivity of the 1st macropores, while the pore size decreased. Foam is a thermodynamically unstable system. In liquids, gas can typically be dispersed into fine bubbles. However, due to their high surface energy and low density, the gases will rise and escape from the liquid surface. The surfactants reduced the gas-liquid interfacial tension and formed a double layer of adsorption on the liquid film of the bubbles, inhibiting the thinning and rupture of the bubbles and reducing the escape of the bubbles. Through the mutual attraction between the surfactant and lipophilic groups, the strength of the double-layer adsorption film and the viscosity of the liquid in the liquid film increased, increasing the stability of the foam system [38,39].

Table 3 shows the properties of the SiC porous ceramics with different amounts of SDS. It can be seen that the addition of SDS increases the porosity and decreases the flexural and compressive strength of SiC porous ceramics.

## 4. Conclusions

(1) In this work, SiC porous ceramics with homogeneous and tunable pore structures were fabricated by the foaming method. Interestingly, there were two series of macropores in the obtained porous ceramics, namely, large pore structures with pore sizes between 0.5 and 3 mm and small pore structures with pore sizes of about 2 μm. The primary phase of SiC porous ceramics was SiC.

(2) With the increase of H_2_O_2_ additions, the porosity, 1st mode pore size, and pore structure connectivity increased, while the bulk density and mechanical strength decreased. The optimal mechanical properties of the SiC porous ceramics were obtained by adding 5 wt.% H_2_O_2_, and the resultant porosity, bulk density, flexural strength, and compressive strength were 56.32%, 2.8301 g/cm^3^, 11.94 MPa, and 24.32 MPa, respectively. SiC porous ceramics showed excellent resistance to acid/alkali corrosion, with only 0.83–3.85% loss of strength after acid/alkali corrosion.

(3) As the foaming temperature increased, the porosity increased, and the mechanical strength decreased. When the foaming temperature was 85 °C, a homogeneous pore structure was obtained. Surfactants were beneficial for improving pore connectivity and porosity.

## Figures and Tables

**Table 1:** Strength loss rate of SiC porous ceramics after acid/alkali corrosion.

Amount of H_2_O_2_ (wt.%)	Strength Loss Rate of SiC Porous Ceramics after Acid Corrosion (%)	Strength Loss Rate of SiC Porous Ceramics after Alkali Corrosion (%)
5	0.83 ± 0.15	1.25 ± 0.14
10	0.91 ± 0.23	1.36 ± 0.38
15	1.88 ± 0.35	2.50 ± 0.27
20	2.00 ± 0.42	2.67 ± 0.34
25	3.08 ± 0.29	3.85 ± 0.26

**Table 2:** Effects of foaming temperature on the properties of SiC porous ceramics with 7.5 wt.% H_2_O_2_ addition.

Foaming Temperature (°C)	Bulk Density (g/cm^3^)	Porosity (%)	Flexural Strength (MPa)	Compressive Strength (MPa)
75	2.71 ± 0.09	45.85 ± 2.69	11.39 ± 1.85	25.37 ± 2.41
85	2.62 ± 0.08	60.22 ± 3.98	9.03 ± 1.42	21.76 ± 1.94
95	2.02 ± 0.05	64.24 ± 2.01	7.01 ± 1.33	20.43 ± 2.12

**Table 3:** The properties of SiC porous ceramics with different amounts of SDS.

SDS Amounts (%)	Porosity (%)	Flexural Strength (MPa)	Compressive Strength (MPa)
0	43.51 ± 2.45	11.28 ± 2.07	26.75 ± 3.47
0.1	60.22 ± 3.98	9.03 ± 1.42	21.76 ± 1.94

## Data Availability

No new data were created or analyzed in this study. Data sharing did not apply to this article.
